# Influence of cavity depth and restoration of non-carious cervical root lesions on strain distribution from various loading sites

**DOI:** 10.1186/s12903-020-01083-w

**Published:** 2020-04-07

**Authors:** Je-Kang Du, Ju-Hui Wu, Ping-Ho Chen, Pei-Shan Ho, Ker-Kong Chen

**Affiliations:** 1 School of Dentistry, Kaohsiung Medical University, 100 Shih-Chuan 1st Road, San-Ming, District, Kaohsiung, 807 Taiwan; 2 Department of Dentistry, Kaohsiung Medical University Hospital, Kaohsiung, 807 Taiwan; 3 Department of Oral Hygiene, College of Dental Medicine, Kaohsiung Medical University, Kaohsiung, 807 Taiwan; 4 Division of Medical Statistics and Bioinformatics, Department of Medical Research, Kaohsiung Medical University Hospital, Kaohsiung, 807 Taiwan

**Keywords:** Non-carious cervical lesion, Defect depth, Strain, Occlusal load, Resin composite

## Abstract

**Background:**

We aimed to investigate the load-induced strain variation in teeth with unrestored and resin-based composite restored non-carious cervical lesions (NCCLs).

**Methods:**

Twelve extracted premolars were provided for measuring buccal-side root NCCLs. Strain gauges were fixed at four measuring sites of each tooth, two at the buccal surface and two at the lingual surface. NCCLs were prepared with occlusal margins at the cemento-enamel junction. A static 9-kg load was applied at seven occlusal loading points: buccal cusp tip (BC), inner inclination of the BC, lingual cusp tip (LC), inner inclination of the LC, center of the mesial marginal ridge or distal marginal ridge, and center of the central groove. The strain was detected at each site in teeth with NCCL depths of 0 (control), 0.5, 1.0, and 1.5 mm. Each NCCL was restored using an adhesive composite resin, and the strains were re-measured.

**Results:**

The strains at the NCCL occlusal and gingival margins decreased with increasing defect depths, and the effect was significant when the depth of the defect was 1.5 mm. Loading on the buccal and lingual cusps induced prominent strain variation. The strains at all depth distribution recovered to nearly intact conditions when the NCCLs were restored.

**Conclusions:**

NCCLs at 1.5 mm depth are detrimental, but they can be restored using resin composites.

**Clinical significance:**

The existence of NCCLs should not be ignored. The depth of the NCCL may affect the progression of the lesion. Resin composite restoration is an appropriate method for preventing persistent NCCL deterioration.

## Background

Non-carious cervical lesions (NCCLs) are wedge-shaped defects frequently observed in the cervical region of a tooth, particularly on the buccal surface [1, 2]. These defects can be seen on any teeth from the canines to molars, but are often seen on the premolars [3–5]. In addition to toothbrush abuse [6, 7] and/or acidic erosion [8–10], NCCLs can be induced by abnormal occlusal forces [11–13] or by a combination of the conditions mentioned above [5, 14].

Regardless of the etiology of an NCCL, a tooth with NCCL continues to sustain persistent and steady occlusal forces, which might have an influence on the further progression of the defect during mastication [15]. The accompanied occlusal force-induced stresses, mainly tensile stresses, are known to be concentrated in the cervical region where an NCCL is apparent [11, 15–19]. Tensile stresses cause the disruption and dissolution of hydroxyapatite during the abnormal occlusal load-induced deformation of the tooth and is postulated to be positively related to the formation of NCCLs [11, 20].

The incidence and severity of NCCLs are known to have a strong correlation with age [3, 21, 22]. The numbers and dimensions of such defects also increase with age [23]. Prolongation of the human life span necessitates an increase in the longevity of the ability of teeth to continue their inherent function, mastication. However, even if NCCLs exist in the remaining teeth, they would be subjected to occlusal loads. Pintado et al. reported the results of a long-term clinical observation study and concluded that occlusal force is positively correlated with the presence of NCCLs [24]. This finding suggests the possibility of an exaggerated influence of occlusal forces on teeth with NCCLs. As NCCLs worsen over time, it is important to investigate the relationship between the presence of an NCCL and its depth to understand whether an increase in NCCL depth has any detrimental effect on the tooth.

The treatment of NCCLs with depths less than 1 mm is usually follow-up at regular intervals [25]. Many reports have mentioned that resin-based composites and resin-modified glass ionomer cement are acceptable for restoring such defects [1, 25–32]. Resin-based composites are more strongly recommended due to their good bonding abilities, satisfactory tooth-colored appearance, and ease of manipulation [31, 32]. However, we found no publication reporting the optimum depth of an NCCL for the restoration to prevent damage or the suitability of resin-based composites in inhibiting NCCL progression.

This study sought to mimic the oral environment and located NCCLs induced by occlusal forces using a strain gauge to measure the strain alteration around the NCCL. We examined NCCLs of 0 mm, 0.5 mm, 1.0 mm, and 1.5 mm depths to determine the influence of NCCL depth on teeth. Furthermore, NCCL of all depths were restored with adhesive and resin-based composite to evaluate the recovery efficacy. The null hypothesis of this study was that a change in the depth of an NCCL influences the strain around the NCCL, and restoration with a resin composite can alleviate the altered strain and restore the tooth to its original condition.

## Methods

Twelve human maxillary first premolars were used in this study. The premolars were extracted for orthodontic purposes and were stored in 2% chloramine T solution. The teeth were examined by using an optical microscope and transillumination examination methods to detect the presence of cracks, fracture lines, or defects. Teeth that revealed any fracture lines or defects on the surface were excluded from the study. The teeth were approximately similar in size with no cracks, caries, or cervical defects. This study was approved by the Institutional Review Board of Kaohsiung Medical University Chung-Ho Memorial Hospital (ID:KMUHIRB-E(I)-20,150,144). The IRB waived the need for informed consent in this study. All experiments were performed in accordance with relevant guidelines and regulations.

### Establishment of strain gauges

**Fig. 1:**
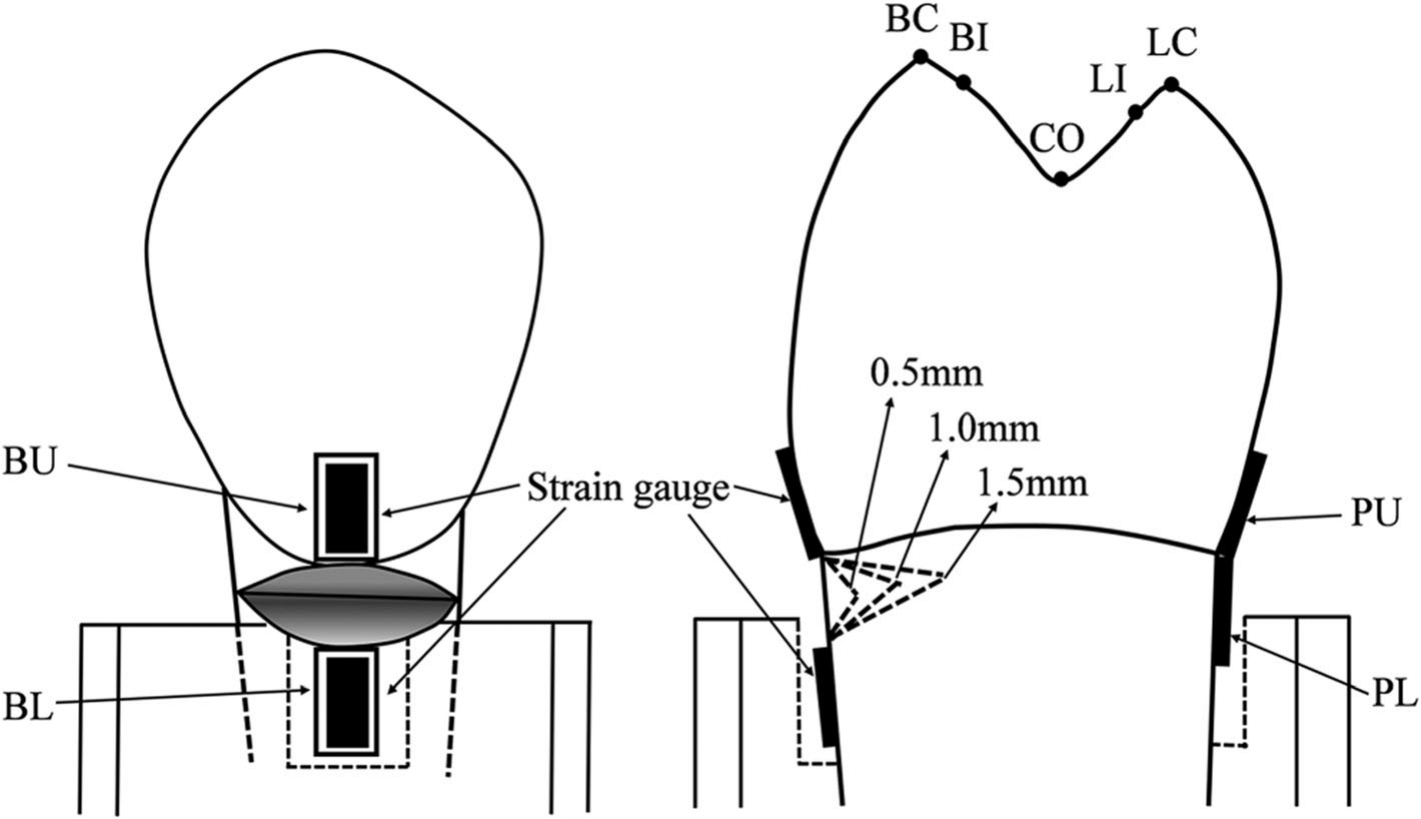
An illustration of NCCL depths, adhesion sites of strain gauges, and occlusal loading sites examined in this study. NCCL: Non-carious cervical lesion

Four strain gauges (KFG-1 N-120-C1-11L1M2R, Kyowa, Japan), two at the buccal surface and two at the lingual surface, were fixed parallel to the tooth axis using a cyano-acrylate base cement (CC-33A, Kyowa, Tokyo, Japan) and were connected to strain amplifiers (DPM-711B, Kyowa, Tokyo, Japan) and a Power Lab (Power Lab/8sp, AD Instruments, CA, USA) for recording the strain variation during loading (Fig. 1).

These strain gauges were located as follows: (1) BU: a strain gauge was fixed to the coronal bucco-cervical region with the tip at the cemento-enamel junction (CEJ) (considered as the occlusal margin of the NCCL); (2) BL: the strain gauge was fixed at the root bucco-cervical region with the tip 1.5 mm from the CEJ (considered as the gingival margin of the NCCL); (3) PU: the strain gauge was fixed at the coronal palato-cervical region with the tip at the CEJ; (4) PL: the strain gauge was fixed at the root palato-cervical region with the tip at the CEJ.

### Occlusal force loading positions

**Fig. 2:**
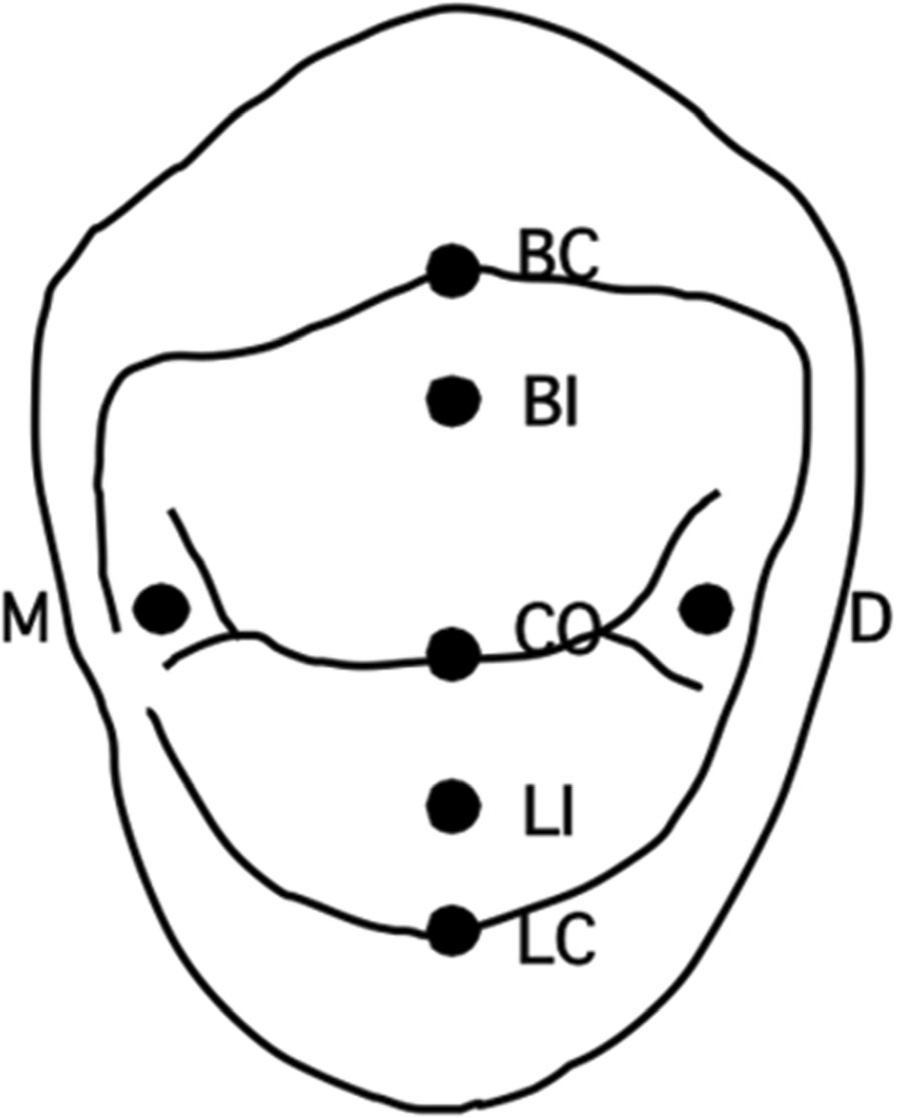
The seven loading points on the occlusal surface

Each root was embedded up to a depth of 2 mm from the CEJ in Type IV Dental stone (high strength) (Fujirock, GC, Tokyo, Japan) in a metal ring. The tooth-ring combination was fixed and prepared to contact with a sharp cone-shaped stainless-steel stylus to receive a static load exerted by a modified servohydraulic testing machine (Model 858, Seiki, Tokyo, Japan). The stylus contact point was kept within 0.1 mm. Seven occlusal loading points were selected based on previous reports [17, 18] as follows: (1) BC: tip of the buccal cusp; (2) BI: point on the buccal triangular ridge, 1 mm lingual to the buccal cusp tip; (3) LC: tip of the lingual cusp; (4) LI: point on the lingual triangular ridge, 1 mm buccal to the lingual cusp tip; (5) CO: point at the center of the central developmental groove; (6) M: point 1 mm distal to the deepest point on the mesial marginal ridge; (7) D: point 1 mm mesial to the deepest point on the distal marginal ridge (Fig. 2). A static load of 9 kg was applied on all points for 3 s for measuring the strains at the four measurement sites simultaneously [17, 18]. The force applied at each point was parallel to the tooth axis. However, the force at BI or LI was applied vertically to each triangular ridge by inclining the tooth-ring combination 45 degrees buccally or lingually, respectively.

### Strain measurement

Strain measurements obtained from the four measurement sites of an intact tooth, NCCL (0), served as baseline measurements (control group). A thin and sharp explorer tip with 0.5 mm calibrations within 1.5 mm length was used for the measurements of depths. After strain measurements at NCCL (0), a V-shaped NCCL (0.5) with dimensions of 1.5 ± 0.1 mm occluso-cervically, 4.0 ± 0.1 mm mesio-distally, and 0.5 ± 0.1 mm bucco-pulpally (depth) was prepared by a tapered diamond bur (#304, Shofu, Kyoto, Japan) with water cooling. The strains on the NCCL (0.5) teeth were measured using the same procedure as used for the NCCL (0) teeth. Once the strains on unrestored NCCL (0.5) were measured, the cavity was treated with a self-etch adhesive, Liner Bond II ∑ (Kuraray, Tokyo, Japan) according to the manufacturer’s instructions and was restored with Clearfil AP-X (A2 shade, Kuraray, Tokyo, Japan) with 40 s of light-curing (550 mW/cm^2^, New Light VL-II, GC, Tokyo, Japan). The resin composite was finished and polished 30 min after light-curing with white stone and CompoMaster (Shofu, Kyoto, Japan) in sequence with water-cooling. The strains on restored NCCL (0.5) teeth were measured. The restored resin composite was removed carefully under a microscope (4X magnification) using the same cone type tapered diamond bur to avoid removal of tooth structure. Presence of residual resin composite was checked by scraping with a sharp explorer tip and was removed carefully and repeatedly until the appearance of intact tooth surface under a microscope (4× magnification). After removal of the resin composite, the cavity was re-measured using the calibrated explorer and a digital caliper (CD-S20C, Mitutoyo, Kawasaki, Japan) to verify compliance with the NCCL dimension requirements. The cavity preparation method described above was repeated, and the depth was increased to 1.0 mm in the unrestored NCCL (1.0) teeth for strain measurements. The strain on resin composite restored-NCCL (1.0) was sequentially measured after the application of the adhesive and resin composite to the NCCL (1.0) teeth. Strain measurements on unrestored-NCCL (1.5) and resin composite restored-NCCL (1.5) teeth were also performed after the NCCL depth was increased to 1.5 mm.

### Statistical analysis

The statistical analyses were performed using SPSS, version 19.0 (Armonk, NY: IBM Corp.). The strain data were analyzed using two-way analysis of variance (ANOVA). The effect of four depths is considered on the strain measurement under adjusting various NCCL locations. Post-hoc comparison of Tukey’s pairs comparison with a significance level of 0.05 was performed to assess the effects of four depths by each of seven loading points. The depths of each composite resin restored-NCCL and NCCL (0) were compared separately.

## Results

**Fig. 3:**
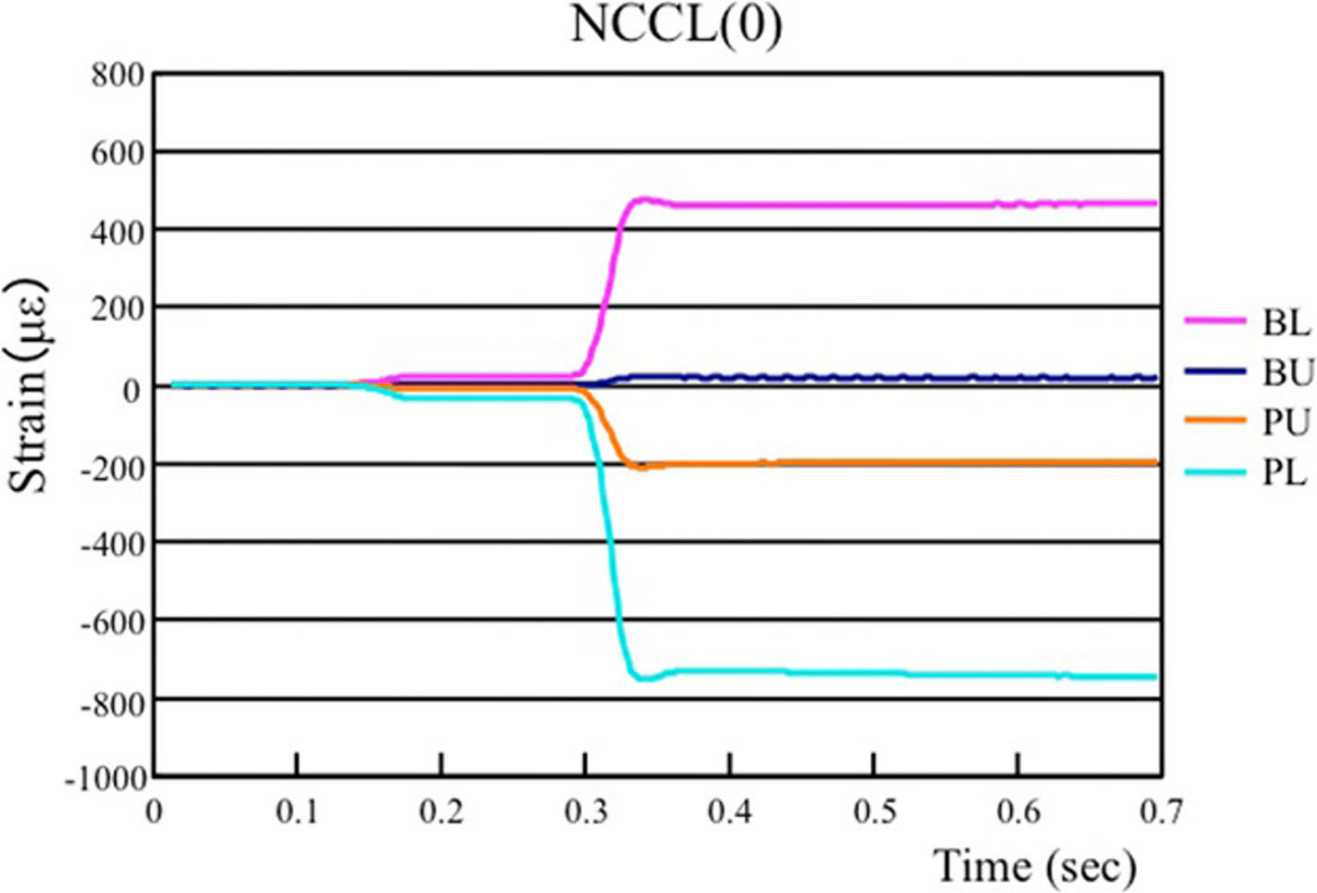
Representative figure showing strain distribution at four sites when a load is applied at the LI loading point on a tooth with no NCCL. NCCL: Non-carious cervical lesion

The results shown in Fig. 3 are representative of the strains on the four measuring sites of an NCCL (0) tooth under LI loading, and the strain patterns demonstrate the presence of tension at the NCCL sites (BU and BL) and of compression at the non-NCCL site (PU and PL).

The maximal strain values at each NCCL measurement site detected at each defect depth and loading point are listed in Table 1. Independent of the depth of the NCCL, the strain values at BL were significantly higher compared to those at BU (*p* < 0.05).

**Table 1:** The comparison of strains measured (Average ± SD) at BU, BL, PU, and PL sites after loading 9 kg at each loading point on teeth with NCCLs of various depths

		Unrestored NCCL site			
Loading point	Depth	Measurement site			
		BU	BL	PU	PL
LC	0 mm	57.89 ± 22.02	159.44 ± 60.82	− 251.33 ± 101.98	− 447.44 ± 99.91
	0.5 mm	47.89 ± 19.96	162.22 ± 70.09	− 243.00 ± 97.31	− 438.22 ± 104.30
	1 mm	35.11 ± 17.83	146.78 ± 64.38	− 249.56 ± 106.94	− 463.56 ± 118.87
	1.5 mm	20.11 ± 17.24	92.44 ± 48.37	− 250.22 ± 110.45	− 453.00 ± 100.31
	*P* value	< 0.01*	0.08	0.10	0.97
		0 mm > 0.5 mm > 1.5mm^a^ 0 mm > 1 mm > 1.5mm^a^			
LI	0 mm	90.56 ± 18.508	514.67 ± 110.89	− 305.89 ± 107.10	− 792.44 ± 110.12
	0.5 mm	68.78 ± 21.534	420.22 ± 128.69	− 279.11 ± 136.24	− 699.22 ± 141.63
	1 mm	47.11 ± 18.286	379.44 ± 85.86	− 294.44 ± 112.93	−759.44 ± 102.09
	1.5 mm	8.78 ± 20.024	269.89 ± 111.10	− 293.22 ± 110.30	− 784.56 ± 100.39
	P value	< 0.001**	< 0.01*	0.97	0.32
		0 mm > 0.5 mm > 1.5mm^a^ 0 mm > 1 mm > 1.5mm^a^	0 mm > 1.5mm^a^ 0.5 mm > 1.5mm^a^ 1 mm > 1.5mm^a^		
BC	0 mm	− 137.67 ± 7.07	− 231.11 ± 36.29	39.22 ± 28.15	99.22 ± 46.75
	0.5 mm	−133.89 ± 15.17	− 230.11 ± 58.99	47.00 ± 37.67	115.89 ± 63.38
	1 mm	− 103.00 ± 10.32	−172.78 ± 45.63	49.22 ± 39.66	118.56 ± 64.31
	1.5 mm	−70.56 ± 24.83	−94.56 ± 51.72	57.33 ± 36.68	151.33 ± 55.20
	P value	< 0.001**	< 0.001**	0.76	0.30
		0 mm < 0.5 mm < 1.5mm^a^ 0 mm < 1 mm < 1.5mm^a^	0 mm < 0.5 mm < 1.5mm^a^ 0 mm < 1 mm < 1.5mm^a^		
BI	0 mm	− 241.44 ± 23.6	− 640.22 ± 13.36A	131.00 ± 80.29	542.67 ± 28.49
	0.5 mm	−206.44 ± 28.25	− 595.00 ± 82.90B	134.11 ± 82.38	546.67 ± 68.15
	1 mm	− 197.22 ± 51.34	− 539.56 ± 95.86B	136.22 ± 79.68	577.56 ± 49.03
	1.5 mm	−93.78 ± 41.351	− 316.89 ± 124.60C	158.67 ± 80.30	636.33 ± 33.06
	P value	< 0.001**	< 0.001**	0.88	< 0.01*
		0 mm < 0.5 mm < 1.5mm^a^ 0 mm < 1 mm < 1.5mm^a^	0 mm < 0.5 mm < 1.5mm^a^ 0 mm < 1 mm < 1.5mm^a^		
M	0 mm	−18.11 ± 34.97	−71.22 ± 65.89	−32.44 ± 22.86	− 108.00 ± 28.43
	0.5 mm	−13.22 ± 34.19	−65.11 ± 85.81	− 25.78 ± 25.97	−91.89 ± 40.96
	1 mm	−10.89 ± 25.55	−62.11 ± 67.77	−21.11 ± 24.59	−80.00 ± 41.38
	1.5 mm	2.89 ± 11.57	−36.00 ± 37.71	−24.89 ± 22.91	−76.78 ± 18.01
	P value	0.44	0.69	0.79	0.21
D	0 mm	−1.89 ± 13.66	−75.67 ± 71.41	−3.11 ± 23.98	−55.11 ± 106.91
	0.5 mm	5.78 ± 15.98	−45.11 ± 44.97	−4.67 ± 29.07	−85.78 ± 119.26
	1 mm	17.89 ± 5.18	−27.33 ± 44.23	−10.89 ± 35.04	−105.44 ± 130.93
	1.5 mm	9.44 ± 7.59	−20.89 ± 31.74	−2.56 ± 32.04	−86.44 ± 125.39
	P value	< 0.01*	0.11	0.93	0.85
		0 mm < 1mm^a^			
CO	0 mm	−16.67 ± 16.49	−17.00 ± 63.67	−105.22 ± 83.92	− 185.56 ± 103.63
	0.5 mm	−15.22 ± 24.71	− 14.67 ± 73.56	−93.11 ± 71.90	− 169.67 ± 96.90
	1 mm	−2.78 ± 14.44	8.11 ± 45.96	−88.11 ± 60.87	−188.22 ± 88.48
	1.5 mm	−6.33 ± 10.25	1.11 ± 36.58	−86.67 ± 73.78	− 160.33 ± 89.43
	P value	0.27	0.74	0.95	0.91

*SD* standard deviation, BU a strain gauge was fixed to the coronal bucco-cervical region with the tip at the cemento-enamel junction (CEJ) (considered as the occlusal margin of the NCCL), *BL* the strain gauge was fixed at the root bucco-cervical region with the tip 1.5 mm from the CEJ (considered as the gingival margin of the NCCL), *PU* the strain gauge was fixed at the coronal palato-cervical region with the tip at the CEJ, PL the strain gauge was fixed at the root palato-cervical region with the tip at the CEJ, *NCCLs* Non-carious cervical lesions

^a^: the statistically significant differences (*p* < 0.05) by the post-hoc comparison of Tukey’s pairs comparison

The absolute strain value measured at BU or BL decreased progressively with the increase in NCCL depth. The strain value at BU and BL was significantly smaller in NCCL (0.5) teeth, and it was the lowest in the NCCL (1.5) teeth. However, no significant differences were observed among the strains at PL or PU with the increase in NCCL depth.

**Fig. 4:**
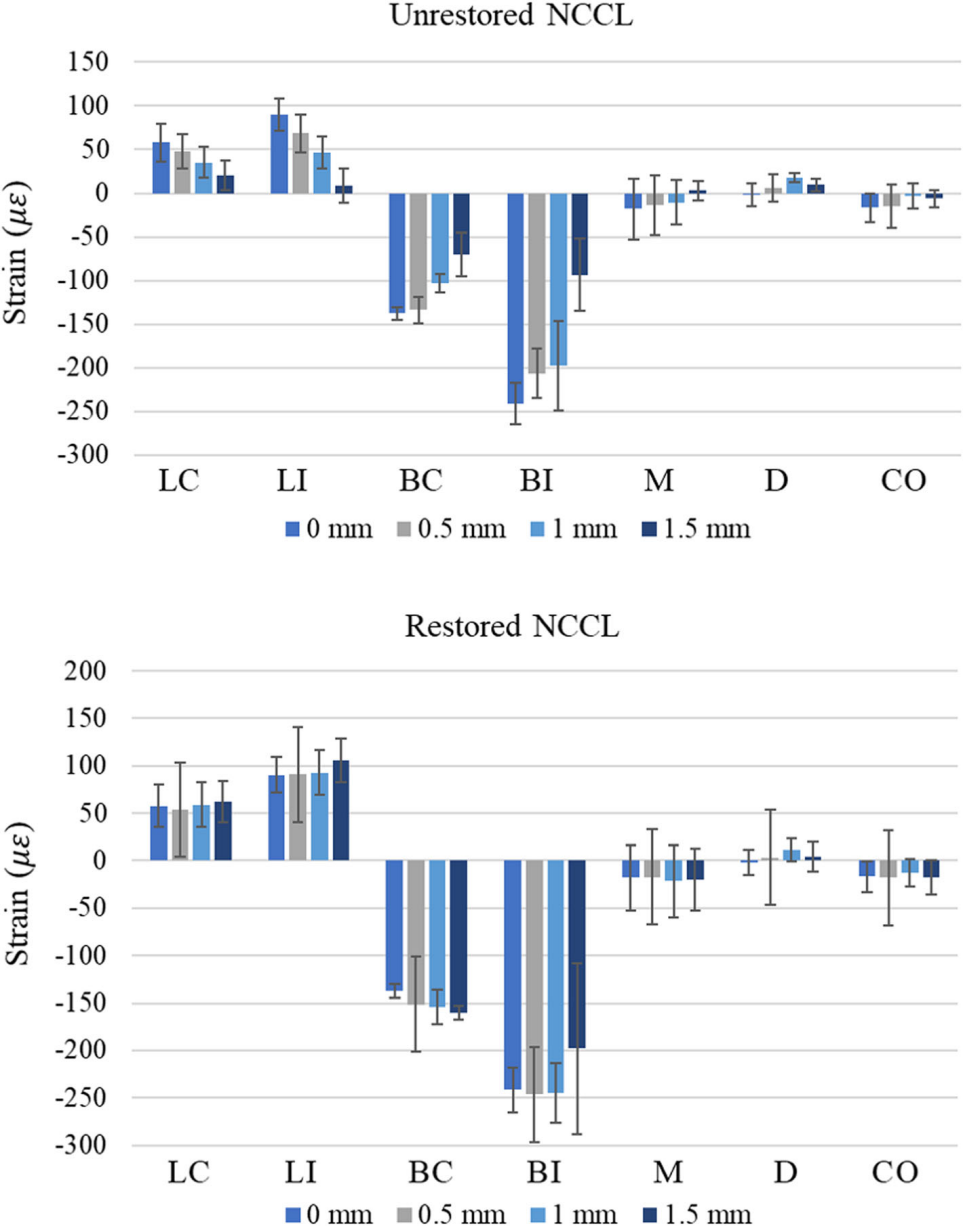
Representative figure showing the strains of unrestored NCCL (upper part) and resin composite restored NCCL (lower part) measured at BU site under different cavity depths and loading points. NCCL: Non-carious cervical lesion

Regardless of NCCL depth, the strains at BU and BL of each restored NCCL tooth showed no significant difference compared with the same sites on NCCL (0) (*p* > 0.05, Fig. 4 and Table 2). Among the restored NCCLs, the strain recovery ratio of NCCL (1.5) tended to be less than that of NCCL (0.5) or NCCL (1.0).

**Table 2:** Strains measured (Average ± SD) at the BU, BL, PU, and PL sites after loading with 9-kg weight at each point on teeth with NCCLs of various depths following restoration with resin composite

		Restored NCCL site			
Loading point	Depth	Measurement site			
		BU	BL	PU	PL
LC	0 mm	57.89 ± 22.02	159.44 ± 60.82	−251.33 ± 101.98	−447.44 ± 99.91
	0.5 mm	53.67 ± 22.40	157.78 ± 61.40	− 236.56 ± 90.55	− 421.33 ± 84.92
	1 mm	59.00 ± 23.60	172.22 ± 63.05	−237.89 ± 94.59	− 433.56 ± 103.34
	1.5 mm	61.89 ± 21.60	174.89 ± 55.66	−237.00 ± 95.75	−412.11 ± 89.46
	P value	0.89	0.90	0.99	0.87
LI	0 mm	90.56 ± 18.51	514.67 ± 110.89	−305.89 ± 107.10	−792.44 ± 110.12
	0.5 mm	90.89 ± 20.48	497.67 ± 97.36	−290.11 ± 118.41	− 770.67 ± 62.24
	1 mm	92.56 ± 23.59	491.89 ± 134.78	−279.00 ± 107.76	− 754.33 ± 120.89
	1.5 mm	105.56 ± 22.98	520.22 ± 106.23	−293.33 ± 110.05	− 733.11 ± 103.20
	P value	0.41	0.94	0.97	0.65
BC	0 mm	−137.67 ± 7.07	−231.11 ± 36.29	39.22 ± 28.15	99.22 ± 46.75
	0.5 mm	− 151.56 ± 22.35	− 238.56 ± 31.77	44.33 ± 31.53	110.56 ± 43.07
	1 mm	− 154.22 ± 17.67	− 240.56 ± 38.33	46.11 ± 35.89	114.00 ± 60.06
	1.5 mm	−160.44 ± 7.18	− 232.00 ± 21.51	43.22 ± 29.69	107.78 ± 44.69
	P value	0.02	0.90	0.97	0.93
BI	0 mm	−241.44 ± 23.62	−640.22 ± 13.36A	131.00 ± 80.29	542.67 ± 28.49
	0.5 mm	− 246.44 ± 42.75	− 632.78 ± 63.97A	133.22 ± 84.09	544.11 ± 75.44
	1 mm	− 244.56 ± 31.52	− 619.56 ± 38.81A	131.44 ± 82.43	544.22 ± 66.36
	1.5 mm	−198.11 ± 90.06	− 532.67 ± 86.55B	136.44 ± 90.57	539.00 ± 110.69
	P value	0.19	< 0.01*	0.10	0.10
			0 mm < 1.5 mm ^a^ 0.5 mm < 1.5 mm ^a^ 1 mm < 1.5 mm ^a^		
M	0 mm	−18.11 ± 34.97	−71.22 ± 65.89	−32.44 ± 22.86	− 108.00 ± 28.43
	0.5 mm	−17.00 ± 31.50	−73.22 ± 79.63	−24.78 ± 23.80	−92.00 ± 43.12
	1 mm	−21.78 ± 38.36	−69.33 ± 79.30	−25.33 ± 28.36	−96.78 ± 45.82
	1.5 mm	−20.11 ± 33.14	−61.11 ± 56.10	−31.22 ± 22.31	−97.67 ± 25.91
	P value	0.99	0.99	0.87	0.83
D	0 mm	−1.89 ± 13.66	−75.67 ± 71.41	−3.11 ± 23.98	−55.11 ± 106.91
	0.5 mm	3.44 ± 19.78	−45.56 ± 38.02	−7.11 ± 25.03	−93.56 ± 112.10
	1 mm	11.44 ± 12.53	−29.89 ± 67.47	−12.33 ± 45.38	− 114.67 ± 155.99
	1.5 mm	3.89 ± 15.78	−31.89 ± 52.02	−10.89 ± 38.79	−95.22 ± 136.22
	P value	0.36	0.34	0.94	0.80
CO	0 mm	−16.67 ± 16.49	−17.00 ± 63.67	− 105.22 ± 83.92	−185.56 ± 103.63
	0.5 mm	−17.89 ± 23.88	−14.11 ± 65.11	−97.22 ± 72.57	− 177.11 ± 84.51
	1 mm	−12.78 ± 14.53	−6.89 ± 57.89	−96.22 ± 83.49	−177.78 ± 91.49
	1.5 mm	−17.67 ± 18.18	−7.44 ± 58.64	−92.67 ± 80.92	− 164.56 ± 94.74
	P value	0.93	0.98	0.99	0.97

*SD* standard deviation, *BU* a strain gauge was fixed to the coronal bucco-cervical region with the tip at the cemento-enamel junction (CEJ) (considered as the occlusal margin of the NCCL), *BL* the strain gauge was fixed at the root bucco-cervical region with the tip 1.5 mm from the CEJ (considered as the gingival margin of the NCCL), *PU* the strain gauge was fixed at the coronal palato-cervical region with the tip at the CEJ, *PL* the strain gauge was fixed at the root palato-cervical region with the tip at the CEJ, *NCCLs* Non-carious cervical lesions

^a^: the statistically significant differences (*p* < 0.05) by the post-hoc comparison of Tukey’s pairs comparison

The LC and LI loading points induced tension strain at the BU and BL sites and compression strain at the PU and PL sites. In contrast, the BC and BI loading points induced compression at the BU and BL sites and tension at the PU and PL sites. When loading at the CO, M, or D points, the strain values were low and were mostly characterized by tension at the BU site and compression at the BL site.

Loading at LC, LI, BC, or BI points resulted in higher strain, and a statistically significant decrease in buccal strain was observed with increased NCCL depth, whereas loading at CO, M, or D points resulted in smaller strains with no marked change, even as the NCCL depth increased to 1.5 mm.

## Discussion

During function, occlusal forces are transmitted through the cervical region toward the root and alveolar bone in premolars and molars. Stresses accompanying occlusal forces are concentrated in the cervical region of the tooth [18, 33–37]. The occlusal force-induced tensile stresses are postulated as the etiology of cervical erosion [11]. A long-term clinical observation has revealed a positive correlation between NCCL progression and occlusal wear [24]. It is conceivable that tooth with NCCL under occlusal loading is accompanied by a risk of magnifying stresses within the NCCL and may lead to the progression of the existing defect and a worsening condition.

In this study, we showed that the strains around occlusal or gingival margins decreased with an increase in NCCL depth regardless of the loading site. Soares et al. and Kuroe et al. have reported that stresses around the margin of an NCCL decrease with the presence of the NCCL and become apparent at the deepest region of the defect [38, 39]. It is conceivable that strains around the cervical third of the root will show the highest value under the conditions of normal morphology, while strain around the NCCL will decrease with the appearance of an NCCL, and the decreased value will be transferred to the inner side of the defect. This may explain why the strain decreased as the NCCL appeared in this study. Table 1 shows a statistically significant difference between NCCL (0) and NCCL (1.5), which indicates the possibility that an NCCL could have a greater influence on the cervical regions of the tooth at a depth of 1.5 mm. In other words, the increasing depth of NCCL results in the concentration of stresses on the pulpal side of the defect, and this could have a progressive detrimental effect, not only on the dentin but also on the pulp. The increasing stresses concentrated at the bottom of an NCCL may be related to the disruption of the crystalline microstructure of the tooth, as proposed by Lee et al. [11], and it may lead to progression of the defect and hypersensitivity [40] or damage to the pulp. NCCLs with depths of 1.5 mm should be considered as borderline for determining whether a conservative restoration should be placed or not.

Regardless of the NCCL depth, different loading sites showed strain fluctuations in this study. The stress was prominent when loading at the buccal or lingual cusp (BI, BC, LI, or LC) and obscure when loading at the proximal marginal ridge or fissure site (M, D, or CO), under the strain used in this study. Another report has mentioned that the cusps exert a bending effect that induces prominent tensile strain at the reciprocal site, while the central pit has a shortening and barreling effect on the crown [41]. However, the marginal ridge deviates from the bucco-lingual line axis with more restrictions from the proximal enamel, leading to a smaller value [34, 42]. The results of strain at the buccal and lingual cusps in this study were consistent with Kuroe’s report [15] indicating that the loading site plays an important role in the strain pattern and magnitude and could exaggerate the stain distribution in NCCLs. Although the tensile strain exerted from LC and LI (considered be related to the formation of abfraction [4, 26, 27, 29, 43, 44]) became smaller with increasing NCCL depth, the persistent appearance of tensile strain from such loading sites may imply the prolonged exertion of destructive stresses on the surrounding hydroxyapatite, which could worsen the configuration of the NCCL.

Both buccal-side strains at each depth measured in both coronal and root NCCLs recovered approximately to the strain values of the intact tooth after resin composite restoration. In particular, the buccal sides of teeth with NCCL (1.5) prominently returned to normal after restoration, with no difference in relation to NCCL location (*p* > 0.05). The importance of restoring NCCL defects instead of ignoring them has been discussed by Grippo in the context of engineering principles [45]. The strain recovery of the resin-restored-NCCL teeth in this study confirmed the necessity of restoration of NCCLs, as proposed by Grippo [45]. The result of our study strongly suggests that an appropriate resin composite restoration of NCCLs could strengthen the weakened tooth to defend against and prevent the formation of deteriorative stresses caused by occlusal loading. In other words, the suitable application of an adhesive/resin composite to an NCCL not only restores the normal morphology and function [32], but also restores the stress distribution to its original condition. This might prevent the further deterioration of the cervical tooth structure and maintain normal tooth function. Therefore, the hypothesis that the depth of an NCCL is an influencing factor and that NCCLs can be treated by resin composite restoration to recover its original condition was supported. Based on our findings, it is recommended that an NCCL deeper than 1.5 mm should be treated aggressively. Failure to address an NCCL of this depth may cause progressive dentin destruction, hypersensitivity, and unexpected pulpitis [46].

There was no periodontal ligament like material used to mimic the clinical condition in this study. Periodontal ligament is known to play an important role of acting as a medium of force transfer during mastication [47]. Therefore, the strain distribution around the cervical region of an NCCL-tooth might be higher than normal in-vivo conditions. It is important to add that the periodontal ligament can inhibit excessive stresses and hence, is necessary for simulating normal clinical conditions. This issue should be considered in future studies. Furthermore, the bonding between the restoration and the tooth was performed on sound dentin of the artificially prepared NCCLs instead of on sclerotic dentin. However, sclerotic dentin is usually observed in the dentinal portion of NCCLs, and the bond strength to sclerotic dentin has been reported to be lower than the bond strength to normal cervical dentin due to the different degree of hypermineralization in sclerotic dentin [48–50]. Further studies addressing the presence of sclerotic dentin in teeth with cervical NCCLs are necessary to examine the strain recovery in NCCL.

## Conclusions

Occlusal force loading induces tensile or compressive strain at the occlusal and gingival sites of NCCLs, depending on the loading site. The deeper the NCCL, the lower the level of strain detected around the defect margins. This phenomenon was significant when the defect extended to a depth of 1.5 mm, but the strain was relieved after restoration of NCCLs of any depth with an adhesive resin composite. Based on our findings, it is recommended that NCCLs with a depth of 1.5 mm should be restored with an adhesive resin to prevent further deterioration of tooth structure and to maintain normal tooth function.

## Data Availability

All data generated or analyzed during this study are included in this published article.

## Abbreviations

NCCLs: Non-carious cervical lesions
CEJ: Cemento-enamel junction
